# Supplementary material for: Analysis of PPARγ Signaling Activity in Psoriasis
Source: Int J Mol Sci. 2021 Aug 10;22(16):8603. doi: 10.3390/ijms22168603 (PMC8395241; doi:10.3390/ijms22168603)
Supplement: Supplementary file 1 [file ijms-22-08603-s001.zip › Supplemental materials_Analysis of PPARg signaling activity in psoriasis/Pathway models/Models images and html files/Differentiation of psoriatic T cells/14116.html]

ZFYVE9


# Protein ZFYVE9

|  |  |
| --- | --- |
| URN | urn:agi-llid:9372 |
| Total Entities | 0 |
| Connectivity | 263 |
| Name | ZFYVE9 |
| Description | zinc finger FYVE-type containing 9 |
| Notes | This gene encodes a double zinc finger motif-containing protein that participates in the transforming growth factor-beta (TGFB) signalling pathway. The encoded protein interacts directly with SMAD2 and SMAD3, and recruits SMAD2 to the TGFB receptor. There are multiple pseudogenes for this gene on chromosomes 2, 15, and X. Alternative splicing results in multiple transcript variants. [provided by RefSeq, Mar 2013] |

---

|  |  |
| --- | --- |
| Pathway | TGF-beta Signaling |
|  | 1\_Differentiation of psoriatic T cells |
|  | Differentiation of psoriatic T cells |

---

|  |  |
| --- | --- |
| MedScan ID | 9372 |

---

|  |  |
| --- | --- |
| LocusLink ID | 9372 |
|  | 230597 |
|  | 313477 |

---

|  |  |
| --- | --- |
| Alias | PPP1R173 |
|  | OTTMUSP00000032178 |
|  | MAD (mothers against decapentaplegic, Drosophila) homolog interacting prote |
|  | zinc finger, FYVE domain containing 9 |
|  | LOC381539 |
|  | MAD, mothers against decapentaplegic homolog interacting prote |
|  | Receptor activation anchor |
|  | LOC277733 |
|  | ZFYVE9 |
|  | MADH interacting protein |
|  | OTTHUMP00000009741 |
|  | Receptor activating anchor |
|  | RP23-406B13.8 |
|  | SARA |
|  | MADH-interacting protein |
|  | SMADIP |
|  | E030027L17 |
|  | MADH-interactive protein |
|  | protein phosphatase 1, regulatory subunit 173 |
|  | AK087111 |
|  | OTTMUSP00000032179 |
|  | zinc finger FYVE-type containing 9 |
|  | Smad anchor for receptor activation |
|  | hSARA |
|  | Mothers against decapentaplegic homolog interacting protein |
|  | LOC230597 |
|  | MAD, mothers against decapentaplegic homolog interacting protein, receptor activation anchor |
|  | OTTHUMP00000009739 |
|  | MAD homolog interacting protein |
|  | zinc finger, FYVE domain containing IX |
|  | OTTHUMP00000009740 |
|  | MADHIP |
|  | OTTMUSP00000008607 |
|  | Mothers against decapentaplegic homolog interactive protein |
|  | NSP |
|  | novel serine protease |
|  | zinc finger FYVE domain-containing protein 9 |

---

|  |  |
| --- | --- |
| GO ID | 0005545 |
|  | 0046872 |
|  | 0019904 |
|  | 0006897 |
|  | 0016197 |
|  | 0007179 |
|  | 0005829 |
|  | 0005769 |
|  | 0031901 |
|  | 0043231 |
|  | 0032991 |
|  | 0046332 |
|  | 0017015 |
|  | 0005737 |
|  | 0008236 |
|  | 0007183 |
|  | 0007184 |
|  | 0006508 |

---

|  |  |
| --- | --- |
| KEGG ID | hsa:9372 |

---

|  |  |
| --- | --- |
| Organism | Homo sapiens {Organism urn:agi-taxid:9606} |
|  | Mus musculus {Organism urn:agi-taxid:10090} |
|  | Rattus norvegicus {Organism urn:agi-taxid:10116} |
|  | Homo sapiens |
|  | Mus musculus |
|  | Rattus norvegicus |

---

|  |  |
| --- | --- |
| Mouse chromosome position | 4 |

---

|  |  |
| --- | --- |
| OMIM ID | 603755 |

---

|  |  |
| --- | --- |
| Rat chromosome position | 5q34 |
|  | 5q35 |

---

|  |  |
| --- | --- |
| Hugo ID | 6775 |

---

|  |  |
| --- | --- |
| Human chromosome position | 1p32.3 |

---

|  |  |
| --- | --- |
| Swiss-Prot Accession | O95405 |
|  | O95405.2 |
|  | A2A8R0 |
|  | A0S860 |
|  | A8Y5G5 |
|  | Q5T0F6 |
|  | Q5T0F7 |
|  | Q9UNE1 |
|  | Q9Y5R7 |

---

|  |  |
| --- | --- |
| GenBank ID | NC\_000001 |
|  | XM\_017002845 |
|  | XP\_016858334 |
|  | NM\_007324 |
|  | NP\_015563 |
|  | NM\_004799 |
|  | NP\_004790 |
|  | XM\_011542437 |
|  | XP\_011540739 |
|  | AC105754 |
|  | AL139156 |
|  | AL513218 |
|  | CH471059 |
|  | EAX06790 |
|  | EAX06791 |
|  | EAX06792 |
|  | HF583785 |
|  | CCQ43282 |
|  | AB209125 |
|  | BAD92362 |
|  | AF104304 |
|  | AAC99462 |
|  | AF130419 |
|  | AAD31694 |
|  | AF130420 |
|  | AAD31695 |
|  | BC032680 |
|  | AAH32680 |
|  | DR005493 |
|  | O95405 |
|  | NC\_000070 |
|  | XM\_006502988 |
|  | XP\_006503051 |
|  | XM\_006502986 |
|  | XP\_006503049 |
|  | XM\_017320143 |
|  | XP\_017175632 |
|  | XM\_006502989 |
|  | XP\_006503052 |
|  | XM\_006502987 |
|  | XP\_006503050 |
|  | NM\_183300 |
|  | NP\_899123 |
|  | NM\_001347163 |
|  | AL607070 |
|  | AL626783 |
|  | AL671909 |
|  | CH466527 |
|  | EDL30733 |
|  | AK081320 |
|  | AK087111 |
|  | BAC39805 |
|  | AK135219 |
|  | BAE22464 |
|  | BC038255 |
|  | BC046341 |
|  | AAH46341 |
|  | BC058416 |
|  | AAH58416 |
|  | BC079578 |
|  | BC094578 |
|  | DQ303413 |
|  | ABC18000 |
|  | NC\_005104 |
|  | NM\_001107952 |
|  | NP\_001101422 |
|  | AC\_000073 |
|  | AABR07049553 |
|  | AABR07049554 |
|  | AAHX01039043 |
|  | CH474008 |
|  | EDL90393 |
|  | EDL90394 |
|  | NC\_018912 |
|  | AMYH02001011 |
|  | AMYH02001012 |
|  | AMYH02001013 |
|  | AMYH02001014 |
|  | AMYH02001015 |
|  | AMYH02001016 |
|  | AMYH02001017 |
|  | AMYH02001018 |
|  | EU176568 |
|  | ABW03369 |
|  | AC\_000026 |
|  | AAHY01038999 |
|  | AAHY01039000 |
|  | AAHY01039001 |
|  | AAHY01039002 |
|  | AAHY01039003 |
|  | AAHY01039004 |
|  | BC156514 |
|  | AAI56515 |
|  | BC172690 |
|  | AAI72690 |

---

|  |  |
| --- | --- |
| Swiss-Prot ID | ZFYV9\_HUMAN |

---

|  |  |
| --- | --- |
| Cell Localization | Cytoplasm |
|  | Early endosome membrane |

---

|  |  |
| --- | --- |
| Ensembl ID | ENSG00000157077 |
|  | ENSP00000287727.3 |
|  | ENST00000287727.7 |
|  | ENSP00000349737.2 |
|  | ENST00000357206.6 |
|  | ENSMUSG00000034557 |
|  | ENSMUSP00000039852.7 |
|  | ENSMUST00000042185.7 |
|  | ENSMUSP00000102269.1 |
|  | ENSMUST00000106658.7 |
|  | ENSMUSP00000102268.1 |
|  | ENSMUST00000106657.7 |
|  | ENSRNOG00000027183 |
|  | ENSRNOP00000036295.6 |
|  | ENSRNOT00000037523.6 |
|  | ENSP00000287727 |
|  | ENST00000287727 |
|  | ENSP00000349737 |
|  | ENST00000357206 |
|  | ENSP00000360647 |
|  | ENST00000371591 |
|  | ENSMUSP00000039852 |
|  | ENSMUST00000042185 |
|  | ENSMUSP00000102269 |
|  | ENSMUST00000106658 |
|  | ENSMUSP00000102268 |
|  | ENSMUST00000106657 |
|  | ENSRNOP00000036295 |
|  | ENSRNOT00000037523 |

---

|  |  |
| --- | --- |
| MGI ID | MGI:2652838 |

---

|  |  |
| --- | --- |
| RGD ID | 1310856 |

---

|  |  |
| --- | --- |
| Unigene ID | Hs.532345 |
|  | Mm.196776 |
|  | Rn.23612 |

---

|  |  |
| --- | --- |
| Homologene ID | 3527 |

---
